# HBV RNA Predicts the Risk of Off‐Treatment Relapse in Chronic Hepatitis B Patients With NAs Therapy: A Systematic Review and Meta‐Analysis

**DOI:** 10.1111/jvh.70167

**Published:** 2026-03-13

**Authors:** Dong‐Hui Wang, Jia‐Lan Wang, Su‐Wen Jiang, Ai‐Wu Zhou, Meng‐Han Jin, Hao‐Jin Zhang, Shi‐Qi Yang, Shi‐Yang Fan, Ai‐Rong Hu

**Affiliations:** ^1^ Liver Diseases Center, Ningbo No. 2 Hospital Ningbo Zhejiang Province China; ^2^ Cixi Biomedical Research Institute Wenzhou Medical University Wenzhou Zhejiang Province China; ^3^ Ningbo University Health Science Center Ningbo Zhejiang Province China; ^4^ School of Medicine Shaoxing University Shaoxing Zhejiang Province China

**Keywords:** chronic hepatitis B, clinical relapse, HBV RNA, nucleoside analogues, viral relapse

## Abstract

Since there are currently few antiviral drugs that can effectively reduce hepatitis B surface antigen levels, the recurrence rate remains high in patients with chronic hepatitis B (CHB) after discontinuing nucleoside analogues (NAs) treatment. This study aims to provide recommendations for monitoring relapse after treatment cessation, while also elucidating the significance of HBV RNA in predicting relapse in CHB patients. Studies published between 2019 and 2025 were searched using PubMed, Embase, Web of Science, Cochrane Library, CNKI, Wanfang, and Cqvip. There are 21 cohort studies included and 2043 individuals involved. In our study, HBV RNA‐positive individuals had a 1.9‐fold higher viral relapse (VR) rate and a 2.26‐fold higher clinical relapse (CR) rate compared to negative patients (all *p* < 0.0001). Subgroup analyses indicated that HBeAg positive at baseline was associated with higher rates of CR (*p* = 0.006) and no significant correlation between longer follow‐up period following the cessation of NAs therapy (≥ 2 years) and higher VR or CR. For each log10 copies/ml increase in HBV RNA levels at discontinuation, there was a 1.32‐fold increase in VR and a 1.37‐fold increase in CR (all *p* < 0.0001). Our findings evaluated the relationship between HBV RNA status and levels at the time of NAs discontinuation and post‐discontinuation relapse, highlighting HBV RNA as a helpful post‐treatment biomarker for predicting relapse. HBV RNA surveillance is essential for patients discontinuing therapy following NAs, particularly for those who are HBeAg‐positive at baseline.

## 1 Introduction

Hepatitis B virus (HBV), the causative agent of hepatitis B, poses a significant global public health challenge. There are about 316 million chronic hepatitis B (CHB) infected people worldwide, and 820,000 people who have died due to cirrhosis and hepatocellular carcinoma (HCC) according to the World Health Organization (WHO) [1, 2]. Current treatment strategies for chronic hepatitis B (CHB) primarily involve continuous oral medication therapy aimed at increasing long‐term survival, minimising chronic infection‐related complications, and halting disease progression. However, achieving the WHO's target of an 80% cure rate by 2030 can be daunting, given the current 2.6% cure rate for CHB [3, 4, 5].

Persistent HBV infection relies on host HBV covalently closed circular DNA (cccDNA), which is pivotal for CHB treatment. Eradicating cccDNA holds the promise of curing HBV infection, enabling patients to live without ongoing treatment or the threat of viral rebound. Regrettably, no current anti‐HBV drug can eliminate both subviral particles and cccDNA [6, 7, 8]. Long‐term suppressive therapy with nucleos(t)ide analogues (NAs) has been proven safe and effective in preventing cirrhosis and HCC in CHB patients [9, 10]. Nonetheless, maintaining adherence to daily treatment poses challenges, and premature discontinuation can hasten the progression to end‐stage liver disease [11, 12]. Moreover, NAs therapy may lead to adverse effects, including mitochondrial toxicity, nausea, fatigue, headache, bone mineral loss, and abdominal pain [13, 14, 15].

Encouragingly, studies suggest that long‐term NA therapy may reduce the entry of Pre‐genome RNA (pgRNA)‐containing capsids into hepatocyte nuclei and deplete cccDNA reservoirs. Each year of NA therapy may decrease cccDNA quantification by approximately 1 log value, making discontinuation theoretically feasible after long‐term treatment, with monitoring of cccDNA levels aiding in safe discontinuation of NAs [16, 17, 18]. However, liver biopsy's invasiveness, the lack of a standardised cccDNA quantification method, and assay complexity underscore the urgent need for non‐invasive markers to monitor cccDNA levels. Several studies have indicated that serum HBV RNA correlates with cccDNA transcriptional activity, making it a potential biomarker for monitoring cccDNA levels [19, 20, 21]. The role of HBV RNA as a predictor of relapse after end‐of‐treatment (EOT) in CHB patients is currently a focus of clinical research. In most studies, HBV RNA shows utility in predicting both viral relapse (VR) and clinical relapse (CR) [22].

Discontinuation has emerged as a viable treatment option for achieving partial cure. In this study, we conducted a systematic review and meta‐analysis to summarise the role of serum HBV RNA in predicting VR and CR after NAs treatment in CHB patients. These findings aim to inform whether CHB patients can safely discontinue NAs therapy and provide guidance for relapse monitoring functional cure to achieve post‐therapy discontinuation functional cure in clinical practice.

## 2 Methods

This systematic review and meta‐analysis was registered in PROSPERO (ID: CRD42024524377) and conducted following the Preferred Reporting Items for Systematic Reviews and Meta‐Analyses (PRISMA) guidelines [17].

### 2.1 Search Strategy

We systematically searched PubMed, Embase, Web of Science, Cochrane Library, CNKI, Wanfang, and Cqvip from January 1, 2019, to July 1, 2025. Meanwhile, we also utilised a combination of MeSH terms and keywords, including Hepatitis B Virus, Chronic Hepatitis B, Hepatitis B Virus Infection, Chronic, Hepatitis B, Chronic, hepatitis B virus pgRNA, hepatitis B virus RNA, HBV RNA, HBV pgRNA, hepatitis B virus pregenomic RNA and HBV pregenomic RNA. An updated search was conducted up to July 1, 2025. Language restrictions were not applied to this study. Moreover, reference lists of review articles and eligible studies were screened for additional relevant studies. The detailed search strategy is outlined in Table S1.

### 2.2 Inclusion and Exclusion Criteria

All full‐text articles that satisfied the following criteria were included: (i) retrospective or prospective longitudinal design studies, (ii) studies involving CHB patients aged ≥ 18 years, (iii) patients treated with any NAs for ≥ 6 months, (iv) available data on EOT HBV RNA qualitatively or quantitatively, or data reported as OR, RR, or HR, with 95% confidence interval (CI), (v) examination of the association between EOT HBV RNA and relapse after treatment. Exclusion criteria were (i) treatment with other antivirals, (ii) patients combined with viral hepatitis other than Type B and severe cardiovascular and cerebrovascular diseases, (iii) insufficient amount of data, (iv) unpublished studies or duplicates.

### 2.3 Study Selection

An initial literature search was independently conducted by three authors (WDH, HAR, and WJL). They assessed each study's title, abstract, and full text, selecting those that met the predetermined inclusion and exclusion criteria. Subsequently, the lists of selected entries were compared, and all authors participated in the decision‐making process regarding study inclusion or exclusion in case of conflicts in the inclusion evaluation. Only articles meeting the eligibility criteria underwent full‐text screening and data extraction. Studies lacking sufficient data for meta‐analysis were excluded. Duplicate or unpublished studies were also ineligible for inclusion.

### 2.4 Data Extraction and Quality Assessment

From eligible papers, we extracted relevant data including the first author, year of publication, study type, sample size, baseline characteristics (country, age, sex, hepatitis B e antigen (HBeAg) serological status, antivirals, duration of treatment, follow‐up period after withdrawal, and outcomes), and definitions of clinical and virological recurrence.

LK and FY independently assessed the quality of observational studies using the Newcastle–Ottawa scale (NOS) [23]. The NOS assigns scores ranging from 0 to 9 based on criteria related to selection, comparability, and outcome. Studies with a score of 7 or higher were considered high quality, while those with scores of 5 or 6 were rated moderate quality, and those with scores of 4 or lower were deemed low quality (Supporting Information Table: Table S2).

### 2.5 Outcome Measurements

The primary outcome measures were VR, defined as serum HBV DNA levels greater than 2000 IU/mL, and CR, defined as VR plus ALT levels exceedingly twice the upper limit of normal. Given variations of HBeAg serological status before treatment, follow‐up period after withdrawal, and HBV RNA testing method, we predefined subgroup analyses to identify individuals at higher risk of relapse after drug withdrawal. We explored the predictive role of HBV RNA test results (both qualitative and quantitative) in predicting relapse after medication withdrawal.

### 2.6 Statistical Analysis

We conducted a meta‐analysis to compile the RR effects between serum HBV RNA status and levels at drug discontinuation and recurrence after discontinuation. In addition to data available for OR, RR, and HR, studies were included if they reported the following outcomes: the number of individuals with positive and negative HBV RNA at treatment cessation, and the number of individuals in each group who experienced clinical or viral recurrence during follow‐up. Studies that did not provide date were excluded. For studies reporting data as OR, the following formula was used to convert the data to RR: RR = OR / (1—*p* + (*p* × OR)), where p is the risk of outcomes in the control group. Results reported as HR were considered RR [24].

Meta‐analysis was performed using R statistical software version 4.2.3 (meta package). The *I* ^2^ statistic was used to measure the degree of heterogeneity between estimates (i.e., 0%–40% may represent insignificant, 30%–60% may represent moderate heterogeneity, 50%–90% may represent substantial heterogeneity, and 75%–100% may represents considerable heterogeneity) [25]. *p*‐values below 0.05 were considered statistically significant. A fixed effects model was employed if *I* ^2^ ≤ 50%, while a random‐effects model was adopted if *I* ^2^ > 50 which is widely assumed as an indication of significant heterogeneity. Cochran's statistic was used for assessing group differences in subgroup analysis and examining the difference between group‐specific overall effect sizes [26].

Sensitivity analyses involved removing one study at a time and redoing the meta‐analysis to assess findings' stability. Publication bias was examined through funnel plot inspection and Egger's linear regression test, with the trim and fill method used for funnel plot asymmetry adjustment. This comprehensive statistical approach aimed to ensure robust findings and interpretive clarity in evaluating the role of serum HBV RNA in predicting relapse after NAs treatment discontinuation in CHB patients.

## 3 Results

**FIGURE 1:**
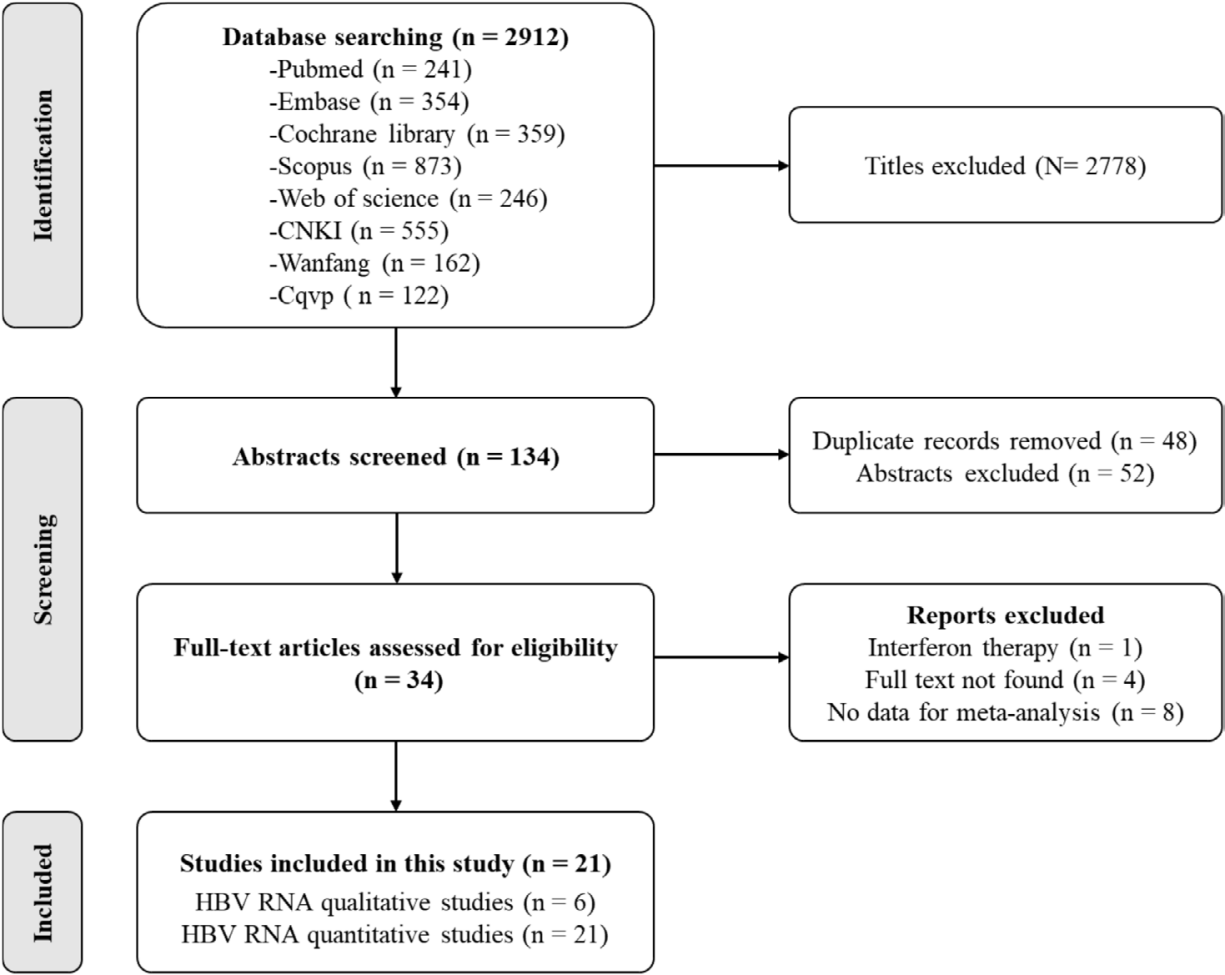
Preferred Reporting Items for Systematic Reviews and Meta‐Analyses flow diagram of study selection.

Our initial searches identified 2912 publications, of which 2778 were excluded based on title screening. After removing 48 duplicates and 52 articles based on abstracts, 34 full‐text articles were assessed for eligibility, leading to the exclusion of 13 articles following full‐text screening. Ultimately, 21 articles (including 23 studies) were included in both qualitative and quantitative synthesis (Figure 1) [20, 27, 28, 29, 30, 31, 32, 33, 34, 35, 36, 37, 38, 39, 40, 41, 42, 43, 44, 45, 46]. All included studies were cohort studies, with subgroups A and B used to distinguish between various studies from the same publication.

### 3.1 Study Characteristics

Table 1 lists all characteristics of the included studies, which comprise 2043 individuals in total. Of the 21 papers, two included only quantitative levels of serum HBV RNA, four included both qualitative [35, 38] and quantitative levels of serum HBV RNA [27, 32, 37, 46], and the remaining 17 included qualitative levels of serum HBV RNA exclusively. Tenofovir disoproxil fumarate (TDF) was the antiviral medication used in one study. TDF or entecavir (ETV) was the antiviral medications used in 6 study populations, telbivudine (LdT) in two studies, and various nucleos(t)ide analogues (NAs) therapy (lamivudine (LAM), adefovir dipivoxil (ADV), LdT, ETV, TDF, and tenofovir alafenamide (TAF)) in the remaining 15 studies.

**TABLE 1:** Study characteristics: Baseline participant characteristics for inclusion in cohort studies.

Reference, year	Country	Study type	Subgroup	Sample size	Age, years	Male gender (*n*, %)	HBeAg status	Duration of treatment	Follow‐up period after withdrawal	Antivirals	Outcome
Apichat Kaew‐dech, 2020 [27]	Thailand	A prospectively study		92	55.0 (50.0–63.0)	59 (64.1)	Positive/negative	6.5 years (5.0–9.5)	142 weeks (126–158)	NAs therapy	CR
Ivana Carey, 2020 [20]	The UK	A retrospectively study	A	23	48	16 (64)	Negative	6.9 years (3.2–11.2)	78 weeks (52–96)	TDF/ETV	CR
		A retrospectively study	B	19	36	11 (58)	Negative	8.5 years (1.6–12.2)	3 months	TDF/ETV	VR
Rong Fan2, Zhou B, 2020 [28]	China	A prospectively study	A	127	30.8 ± 6.9	94 (72.4)	Positive	243.8 weeks	4 years	LdT	VR or CR
		A prospectively study	B	40	36.5 ± 9.4	29 (72.5)	Positive	≥ 2 years	5.5 years	TDF/ETV	VR or CR
Rong Fan, Peng J, 2020 [29]	China	A prospectively study		127	30 (25–35)	92 (72.4)	Positive	4.4 years	4 years	LdT	VR or CR
Yayun Liu, 2020 [30]	China	A prospectively study		29	46 (35.75–59)	21 (70)	Positive/negative	57.7 months (30.25–77.5)	2 years	NAs therapy	VR or CR
Mireia García‐López, 2021 [31]	Spain	A prospectively study		27	56 (45–61)	21 (78)	Negative	≥ 3 years	2 years	TDF/ETV	VR
Muye Xia, 2021 [32]	China	A prospectively study		135	36.1	110 (81.5)	Positive/negative	≥ 2 years	2.6 years (1.0–3.5)	NAs therapy	VR
Wai‐Kay Seto, 2021 [33]	China	A prospectively study		114	58.4 (51.3–65.3)	75 (65.8)	Negative	6.7 years (5.5–7.8)	48 weeks	TDF/ETV	VR or CR
Yandi Xie, 2021 [34]	China	A multicenter prospective cohort study		139	36	81 (58.3)	Positive	6.4 years (4.7–8.6)	2 years	NAs therapy	VR or CR
Yue Liu, 2021 [35]	China	A retrospectively study		142	27.24	106 (74.6)	Positive	12‐89 months	2–96 months	Lamivudine	VR
Apichat Kaew‐ dech, 2022 [36]	Thailand	A retrospectively study		92	55.0 (50.0–63.0)	59 (64.1)	Positive/negative	6.5 years (5.0‐9.5)	3.5 years	NAs therapy y	VR or CR
Andreas Laras, 2022 [37]	Greece	A retrospectively study		74	60 (27–83)	55 (74.3)	Negative	75 months (36–220)	15 months (1‐96)	NAs therapy	VR or CR
Jingyu Chen 2022 [38]	China	A retrospectively study		218	54	116 (53.2)	Negative	≥ 2 years	2 years	NAs therapy	VR
Jinzhi He, 2022 [39]	China	A prospectively study		91	43 (21–66)	63 (69.2)	Negative	≥ 2 years	1 years	NAs therapy	VR
Margarita Papatheodoridi, 2022 [40]	Greece	A prospective twocentre cohort study		57	60 ± 14	37 (65)	Negative	≥ 4 years	1 years	TDF/ETV	VR or CR
Alexander J. Thompson, 2023 [41]	Australia	A prospective, multicenter, observational study		65	56 (31–74)	35 (54)	Negative	≥ 2 years	96 weeks	NAs therapy	VR or CR
Florian van Bömmel 2024 [42]	Germany	A prospectively study		41	47.4 ± 10.55	29 (64.4)	Negative	≥ 2 years	48 weeks	ETV/TDF/TAF	VR or CR
Shuo Wu 2024 [43]	China	A retrospectively study		81	35 (31–44)	50 (61.7)	Positive/negative	65 (40–107) months	32 (24–48) months	NAs therapy	VR or CR
Simon J. Hume 2024 [44]	Australia	A prospective, multicentre study		65	54 (48–60)	35 (54)	Positive/negative	≥ 2 years	96 weeks	NAs therapy	VR or CR
Norah A. Terrault 2024 [45]	The USA	A prospectively study		91	44.2 (39.0–57.7)	60 (65.9)	Negative	192 weeks	48 weeks	TDF	CR
Valerie Ohlendorf 2024 [46]	The Asia Pacific region	A retrospectively, multicentre study		154	52.5 (20–66)	115 (75)	Negative	≥ 2 years	24 weeks	TDF/ETV	VR or CR

Abbreviations: CR, clinical relapse; ETV, entecavir; HBeAg status, hepatitis B e antigen serological status; LdT, telbivudine; NA, nucleos(t)ide analogues; TDF, tenofovir disoproxil fumarate; VR, virological relapse.

In 19 studies, 11 (57.9%) indicated that the risk of viral rebound (VR) was higher in HBV patients with positive HBV RNA at the time of discontinuation compared to those with negative HBV RNA. Eight studies found no statistically significant difference between HBV RNA status and the risk of recurrence after discontinuation of NAs. Among 20 studies, 13 (65%) indicated that the risk of CR was higher in HBV RNA‐positive patients compared to HBV RNA‐negative patients when discontinuing treatment, while the other seven studies found no statistically significant difference between HBV RNA status and the risk of recurrence after discontinuation of NAs.

### 3.2 Qualitative Assessment of HBV RNA

**FIGURE 2:**
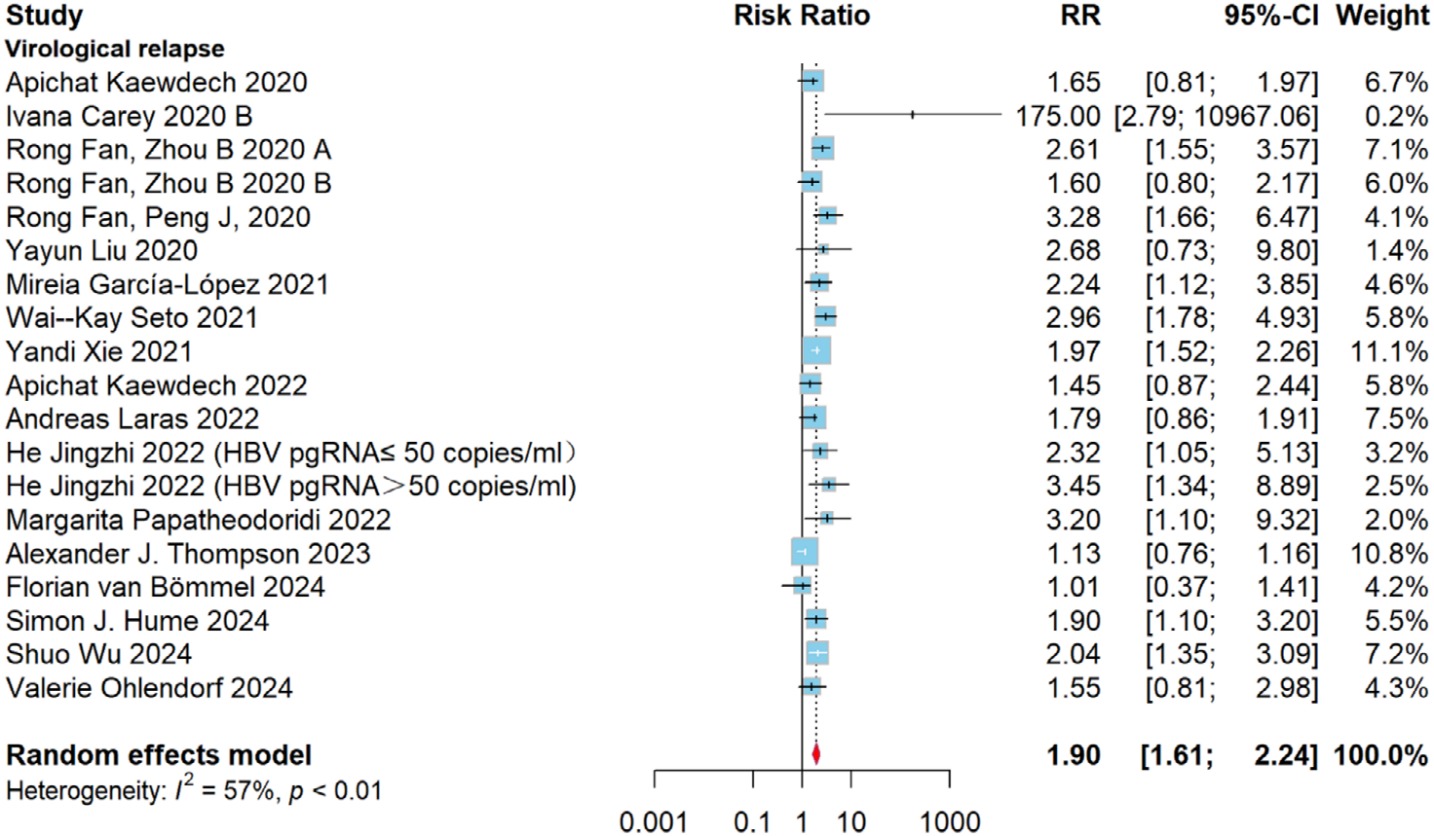
Forest plot of the qualitative VR group: Average effect size of patients with and without HBV RNA at the time of stopping treatment on the RR for recurrence following stopping treatment. Based on 19 cohort studies. Determined with the aid of a fixed‐effects model. Test for overall effect: *Z* = 7.66, *p* < 0.0001. *I* ^2^ = 57%. The weighted mean differences between the HBV RNA Positive and HBV RNA Negative groups are shown by squares. Different sized squares show various weights for various study sample sizes. 95% CI is shown by horizontal lines and parentheses. CI, confidence interval; RR, risk ratio.

In 20 studies, 1901 individuals underwent qualitative testing of serum HBV RNA levels. Those with positive HBV RNA comprised the test group, while those with negative HBV RNA formed the control group. Among them, 1434 individuals with VR as the endpoint measure and RR as the metric used to examine the degree to which HBV RNA positive affects recurrence following medication termination. Figure 2 presents the correlation analysis between HBV RNA status at the time of discontinuation and the risk of VR after discontinuation. When HBV RNA was positive at the time of discontinuation, the risk of VR after discontinuation was 1.90 (95% CI: 1.61–2.24; *I* ^2^ = 57%). The *I* ^2^ test indicated moderate heterogeneity in all analyses. When grouped by HBeAg status, the RR was 2.10 (95% CI: 1.73–2.56; *I* ^2^ = 29%) in HBeAg‐positive patients, 1.87 (95% CI: 1.43–2.45, *I* ^2^ = 65%) in HBeAg‐negative patients, and 1.77 (95% CI: 1.37–2.28, *I* ^2^ = 0%) in HBeAg‐negative/positive patients (Figure S1). Subgroup analysis based on follow‐up duration revealed that the pooled effect size for studies with a follow‐up duration of ≥ 2 years was 1.99 (95% CI: 1.74–2.28, *I* ^2^ = 0%), while that for studies with a follow‐up duration of < 2 years was 1.85 (95% CI: 1.38–2.48, *I* ^2^ = 67%). Although the heterogeneity decreased, the difference between the two groups was not statistically significant (*p* > 0.05) (Figure S2). Subgroup analysis based on HBV RNA detection methods revealed that the pooled effect size for studies with quantitative polymerase chain reaction (RT‐qPCR) (15 studies) was 1.86 (95% CI: 1.54–2.24, *I* ^2^ = 60.9%), with simultaneous amplification testing (SAT) (3 studies) was 2.34 (95% CI: 1.73–3.29, *I* ^2^ = 0%), while that for a study with droplet digital PCR (dd PCR) was 2.37 (95% CI: 0.87–2.43). Again, although the heterogeneity decreased, the difference between the three groups was not statistically significant (*p* > 0.05).

**FIGURE 3:**
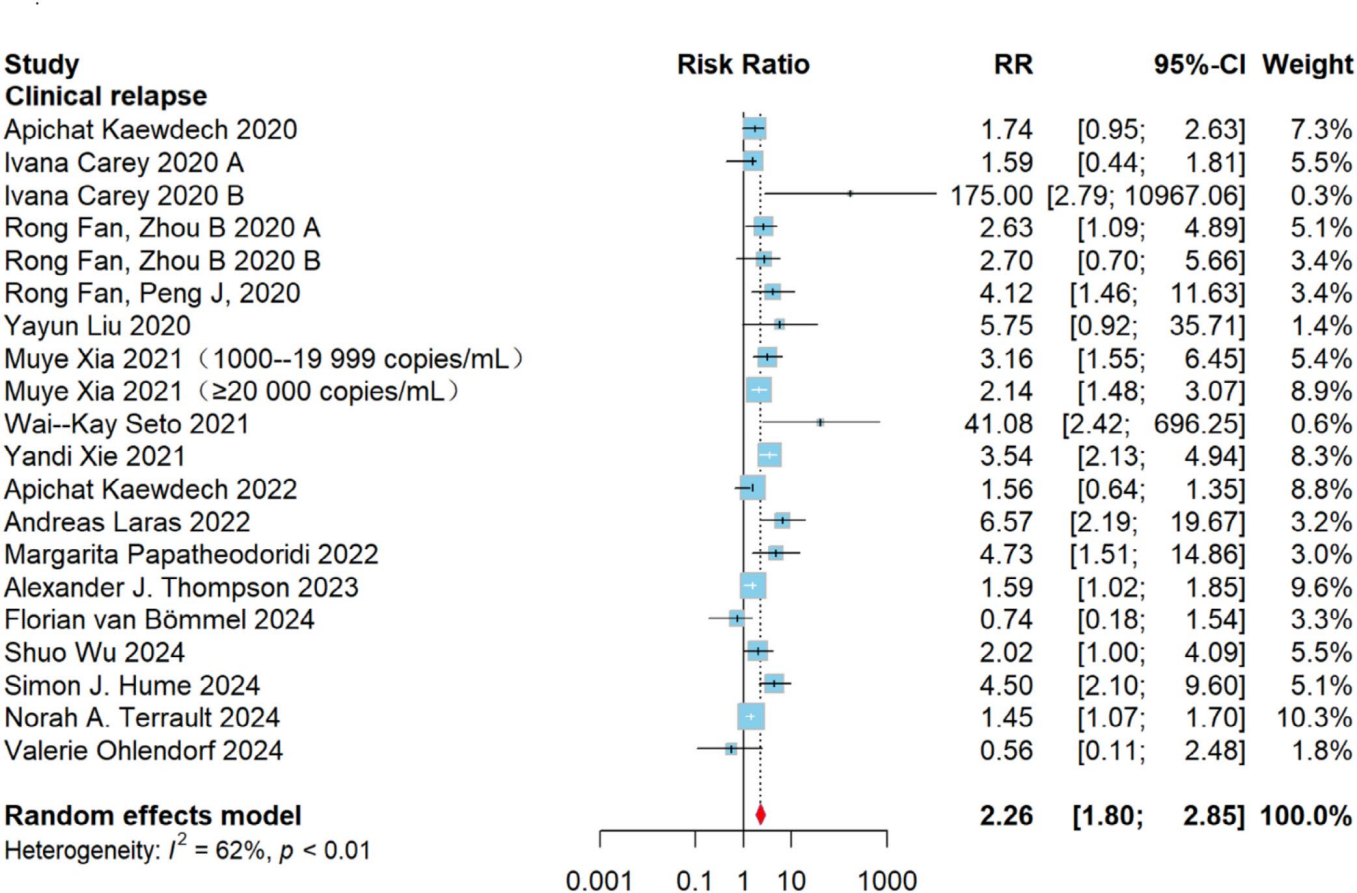
Forest plot of the qualitative CR group: Average effect size of patients with and without HBV RNA at the time of stopping treatment on the RR for recurrence following stopping treatment. Based on 20 cohort studies. Determined with the aid of a fixed‐effects model. Test for overall effect: *Z* = 7, *p* < 0.0001. *I* ^2^ = 62%. The weighted mean differences between the text and control groups are shown by squares. Different sized squares show various weights for various study sample sizes. 95% CI is shown by horizontal lines and parentheses. CI, confidence interval; RR, risk ratio.

In order to determine the degree to which HBV RNA positivity influences recurrence following therapy termination, 1565 patients were assessed using CR as the endpoint and RR as the indicator. The association analysis between the probability of CR following discontinuation and the HBV RNA status at discontinuation is shown in Figure 3. When HBV RNA was positive at discontinuation, the risk of CR after discontinuation was 2.26 (95% CI: 1.80–2.85; *I* ^2^ = 62%). The *I* ^2^ test indicated moderate heterogeneity in all analyses. When grouped by HBeAg status, the RR was 3.30 (95% CI: 2.38–4.59, *I* ^2^ = 0%) in HBeAg‐positive patients, 2.67 (95% CI: 1.31–5.42, *I* ^2^ = 74%) in HBeAg‐negative patients, and 1.82 (95% CI: 1.54–2.16, *I* ^2^ = 2%) in HBeAg‐negative/positive patients. Differences between subgroups were statistically significant (χ^2^ = 10.38, *p* = 0.006) (Figure S3). Subgroup analysis based on follow‐up duration revealed that the pooled effect size for studies with a follow‐up duration of ≥ 2 years was 2.36 (95% CI: 1.85–3.00, *I* ^2^ = 29%), while that for studies with a follow‐up duration of < 2 years was 2.37 (95% CI: 1.34–4.19, *I* ^2^ = 71%). Again, although the heterogeneity decreased, the difference between the two groups was not statistically significant (*p* > 0.05) (Figure S4). Subgroup analysis based on HBV RNA detection methods revealed that the pooled effect size for studies with RT‐qPCR (16 studies) was 2.30 (95% CI: 1.77–2.98, *I* ^2^ = 65%), with SAT (3 studies) was 4.92 (95% CI: 1.09–22.13, *I* ^2^ = 58%), while that for a study with droplet digital PCR (dd PCR) was 1.56 (95% CI: 1.08–2.27). The difference between the three groups was not statistically significant (*p* > 0.05).

### 3.3 Quantitative Assessment of HBV RNA

Six studies were included in this meta‐analysis, examining the relationship between quantitative serum HBV RNA levels and VR and CR in individuals with CHB following NAs treatment termination. Log10 copies/ml was the unit of measurement. Since all data obtained in the study were HR, we combined the indicators as HR. In addition, the HBV RNA detection method used in the institute is RT‐qPCR.

**FIGURE 4:**
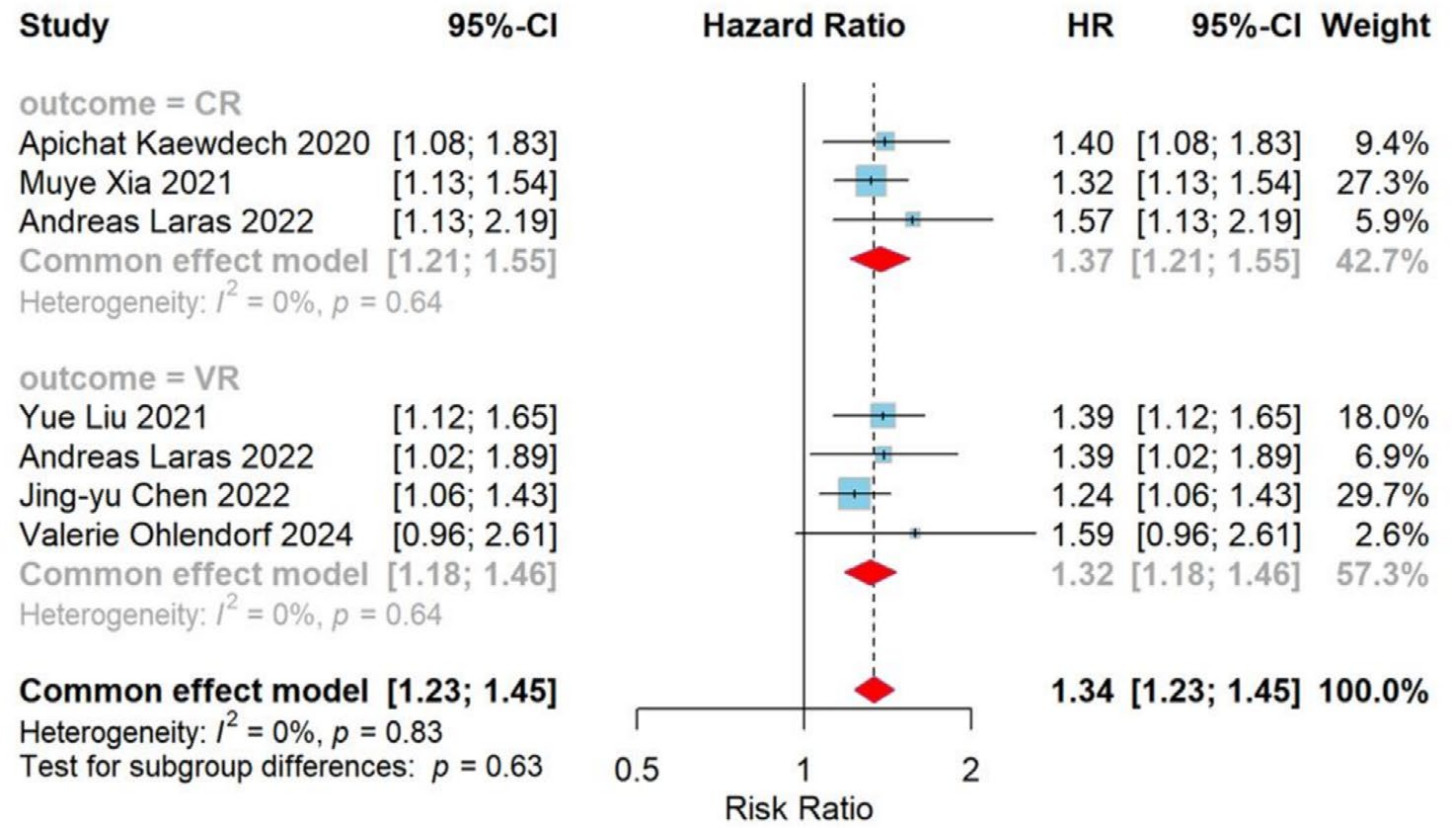
Forest plot of the quantitative HR group: Mean Effect Size for HR of Serum HBV RNA Levels at Discontinuation in the NAs‐Treated Relapsed vs. Non‐Relapsed Population. Based on 7 cohort studies. Determined with the aid of a fixed‐effects model. Test for overall effect: VR: *Z* = 4.98, *p* < 0.0001; CR: *Z* = 5.02, *p* < 0.0001, *I* ^2^ = 0%. The weighted mean differences between the HBV RNA Positive and HBV RNA Negative groups are shown by squares. Different sized squares show various weights for various study sample sizes. 95% CI is shown by horizontal lines and parentheses. CI, confidence interval; HR, hazard ratio.

Serum HBV RNA level testing was performed on 815 participants in 6 studies. The correlation analysis between the probability of recurrence upon cessation and the hepatitis B virus RNA levels at the time of cessation is displayed in Figure 4. For each per log10 copies/ml increase in HBV RNA at the time of discontinuation, the risk of VR after discontinuation was 1.32 (95% confidence interval: 1.18–1.46; *I* ^2^ = 0%), and the risk of CR was 1.37 (95% confidence interval: 1.21–1.55; *I* ^2^ = 0%). The *I* ^2^ test indicated no heterogeneity across analyses. In addition, subgroup analyses were performed based on HBeAg serological status before treatment and follow‐up period after withdrawal, which showed that there were no statistically significant differences between subgroups (all *p* > 0.05) (Figures S5 and S6).

### 3.4 Sensitivity Analyses

The leave‐one‐out sensitivity analyses, displayed in Figures S7–S9, revealed no significant change in effect size when the qualitative VR group, qualitative CR group, and quantitative group were removed from the analysis across various groups (refer to Figures S4–S6). For the qualitative VR group, qualitative CR group, and quantitative group, the effect sizes ranged from 1.61 to 2.24 (*p* < 0.01), 1.8 to 2.85 (*p* < 0.01), and 1.23 to 1.45 (*p* < 0.01). However, when the study carried out by Alexander J. Thompson in 2023 [41] was removed from the qualitative VR group, the heterogeneity changed from moderate (*I* ^2^ = 57%) to lower (*I* ^2^ = 18%) (Figure S7).

### 3.5 Publication Bias

Funnel plots used to test for publication bias revealed missing studies on the left sides for the qualitative VR group, qualitative CR group, and quantitative group (Figures S10, S12, S14). Egger's test supported this finding, indicating the existence of a slight study effect (*p* = 0.027, *p* = 0.008, and *p* = 0.018, respectively). Corresponding trim‐and‐fill plots were generated for these groups (Figures S11, 13, 15).

## 4 Discussion

In order to assess the impact of serum HBV RNA status and levels on post‐drug discontinuation relapse in patients with CHB, a systematic review and meta‐analysis of 21 articles (including 23 cohort studies) were conducted. The findings have revealed a correlation between virologic and clinical recurrence upon discontinuation and EOT HBV RNA positivity. Additionally, the likelihood of relapse increased with higher levels of HBV RNA measured. Prior research lacked comprehensive subgroup analyses and meta‐analyses of serum HBV RNA quantification, limiting insights into participant and study characteristics [47].

After HBV enters hepatocytes, it forms cccDNA primarily in the nucleus, acting as a transcriptional template for all viral transcripts. To keep the cccDNA reservoir full, nucleocapsid‐containing cytoplasmic DNA is recycled back into the nucleus. The challenge lies in eradicating cccDNA completely, contributing to persistent infection and relapse after drug discontinuation [48, 49]. While NAs therapy effectively suppresses HBV replication and reduces serum HBV DNA to undetectable levels, it does not eliminate cccDNA from the liver. Serum HBV DNA levels do not accurately reflect cccDNA levels in infected cell nuclei [50, 51]. HBV cccDNA persistence impedes clinical cure for CHB, with recurrence being more easily observed after treatment is discontinued [52]. Serum HBV RNA, mainly composed of pgRNA, can be released into the peripheral blood after being transcribed using cccDNA and can be reverse transcribed to HBV DNA [18, 19, 53]. This meta‐analysis investigates the link between serum HBV RNA status and level at the time of drug discontinuation and relapse after drug discontinuation.

According to the recommended duration of NAs treatment withdrawal in international guidelines [54, 55, 56], all three guidelines indicate that: patients with non‐cirrhotic HBeAg‐positive CHB who achieve stable HBeAg seroconversion and undetectable HBV DNA for at least 12 months may stop taking NAs. Long‐term (at least 2–3 years) virologic suppression obtained after NAs therapy in non‐cirrhotic HBeAg‐negative patients may allow discontinuation of NAs. Unfortunately, even patients who meet the criteria for discontinuation remain at risk for relapse and hepatitis flares after discontinuation [52]. Reduction in hepatitis B surface antigen (HBsAg) is considered to be the optimal therapeutic endpoint and has been termed a ‘functional cure’. Yet this is rarely accomplished with our current antiviral medication (< 1%) and the annual incidence of spontaneous HBsAg seroconversion has been reported to be approximately 1% [57, 58]. Furthermore, HBsAg's usefulness in predicting cccDNA levels and transcriptional activity is compromised by the possibility that it originates from viral sequences integrated into the host genome, particularly in individuals with HBeAg negative status [59, 60]. Therefore, further new biomarkers might be required to direct the discontinuation of NAs.

The study demonstrated that among patients who achieved the treatment discontinuation conditions, the presence of HBV RNA in serum at the time of treatment discontinuation was associated with an increased rate of viral recurrence and clinical recurrence. The rate of VR in HBV RNA‐positive individuals was 1.9 times higher than in HBV RNA‐negative patients, and the CR rate was 2.26 times higher in HBV RNA‐positive patients than in HBV RNA‐negative patients (VR: *Z* = 7.66, *p* < 0.0001; CR: *Z* = 7, *p* < 0.0001). Moreover, this study conducted subgroup analyses based on the HBV HBeAg status at baseline and follow‐up period after withdrawal. In subgroup analyses of the qualitative CR group, we found that baseline HBeAg status influenced the predictive efficacy of HBV RNA. Specifically, the relative risk of CR was significantly higher in baseline HBeAg‐positive patients (RR = 3.30) compared to HBeAg‐negative patients (RR = 2.67). This suggests that while HBV RNA can predict recurrence in both patient groups, its predictive strength is more pronounced in HBeAg‐positive patients. Therefore, for patients who are HBeAg‐positive at baseline, achieving HBV RNA seroconversion after meeting discontinuation criteria may be crucial for reducing the risk of post‐treatment recurrence. It is well‐known that the presence of HBeAg signifies persistent HBV infection in the liver tissue. According to earlier research, among HBeAg‐positive CHB patients, HBV RNA may serve as a predictor of spontaneous seroconversion to HBeAg [61]. Furthermore, in HBeAg‐positive patients, serum HBV RNA was positively correlated with HBeAg, and the correlation increased with the duration of drug therapy [62, 63]. However, research on the relationship between HBV RNA and patients who test negative for HBeAg is scarce.

The duration of consolidation therapy and the length of treatment were linked to the risk of post‐discontinuation relapse in earlier research [64]. Our subgroup analysis further revealed that HBV RNA positivity at discontinuation consistently predicted VR and CR regardless of whether follow‐up exceeded 2 years, with no significant difference in predictive strength between subgroups (*p* > 0.05). This demonstrates the robustness of HBV RNA status at discontinuation as a predictive factor. It is well established that post‐discontinuation relapse, particularly severe clinical relapse, predominantly occurs between 6 months and 2 years after treatment cessation. Our data also support close monitoring of patients during this high‐risk period (at least 2 years post‐discontinuation). However, for patients who remain relapse‐free within 2 years post‐discontinuation, although their short‐term risk is reduced, the persistent presence of cccDNA means that the risk of long‐term VR and CR cannot be entirely ruled out. Therefore, long‐term, regular follow‐up remains essential. Subsequent subgroup analysis of HBV RNA detection methods suggested that HBV‐SAT may enhance the predictive strength of HBV RNA, though differences between groups were not statistically significant. This indicates that despite variations in detection methods, their impact on the direction of primary conclusions may be limited. Future studies should further standardise HBV RNA detection methods to improve comparability across research.

When quantitative HBV RNA levels were analysed at the time of discontinuation, the results showed that higher levels of EOT HBV RNA were associated with a higher rate of VR and CR after discontinuation. There was a 1.32‐fold increase in the risk of virologic relapse and a 1.37‐fold increase in the risk of clinical relapse for each log10 copies/ml increase in EOT HBV RNA levels (VR: *Z* = 4.98, *p* < 0.0001; CR: *Z* = 5.02, *p* < 0.0001). Thus, the risk of relapse after discontinuation was positively correlated with EOT HBV RNA levels.

This meta‐analysis had several limitations. Firstly, moderate heterogeneity was observed in the qualitative VR group and the CR group. Nevertheless, given variations in baseline traits, HBV RNA testing method, follow‐up durations, and other factors such as baseline HBeAg status, this variability was not unexpected. Subgroup analysis revealed variations in baseline HBeAg status as a source of heterogeneity. Secondly, although we found that there was no statistically significant difference among studies with follow‐up periods greater than 2 years, the study included 5 studies with follow‐up periods of less than one year, which may have led to an overestimation of the long‐term benefits. Further long‐term trials are needed. Lastly, several studies had relatively small sample sizes, potentially introducing publication bias and affecting result stability. Furthermore, although we explored the impact of detection methods, differences in HBV RNA detection platforms, reagents, and cutoff values across included studies remained. This may represent one potential source of the moderate heterogeneity observed in our analysis. Variations in sensitivity and specificity among different detection methods could affect the precise pooling of results. Future research should focus on advancing the standardisation of HBV RNA detection to more accurately assess its efficacy as a marker for predicting recurrence after treatment discontinuation.

In conclusion, EOT blood HBV RNA levels and status are associated with NAs discontinuation relapse in CHB patients, offering predictive value, especially in patients positive for HBeAg at baseline. Serum HBV RNA serves as a helpful biomarker for predicting clinical recurrence following cessation. To minimise the relapse rate after discontinuation, additional consolidation therapy to achieve HBV RNA‐negativity before withdrawal may be necessary in HBV RNA‐positive patients, especially those who are HBeAg‐positive and meet discontinuation criteria.

## Funding

This work was supported by Major Medical Scientific Research Foundation of China National Health Commission‐Zhejiang Province under Grant No. WKJ‐ZJ‐2341; Ningbo Medical & Health Brand Discipline under Grant No. PPXK2024‐04; Ningbo Public Welfare Research Foundation under Grant No. 2024S027; Hwamei Research Fund of Ningbo No. 2 Hospital (2025HM07) and Zhejiang Province Key Clinical Specialty Construction Project 2024023.

## Conflicts of Interest

The authors declare no conflicts of interest.

## Supporting information


**Table S1:** Search Strategy.
S2 Table: Result of Newcastle‐Ottawa scale quality assessment.

**Figure S1:** Subgroup analysis of the qualitative VR group: Depending on patients' different HBV HBeAg status.
**Figure S2:** Subgroup analysis of the qualitative VR group: Depending on patients' different follow‐up duration.
**Figure S3:** Subgroup analysis of the qualitative CR group: Depending on patients' different HBV HBeAg status.
**Figure S4:** Subgroup analysis of the qualitative CR group: Depending on patients' different follow‐up duration.
**Figure S5:** Subgroup analysis of the quantitative group: Depending on patients' different HBV HBeAg status.
**Figure S6:** Subgroup analysis of the quantitative group: Depending on patients' different follow‐up duration.
**Figure S7:** Sensitivity analysis of effect on the qualitative VR group‐ leave‐one‐out analysis.
**Figure S8:** Sensitivity analysis of effect on the qualitative CR group‐ leave‐one‐out analysis.
**Figure S9:** Sensitivity analysis of effect on the quantitative group‐leave‐one‐out analysis.
**Figure S10:** Funnel plot of the effect on the qualitative VR group.
**Figure S11:** Trim and fill plot for the effect on the qualitative VR group.
**Figure S12:** Funnel plot of the effect on the qualitative CR group.
**Figure S13:** Trim and fill plot for the effect on the qualitative CR group.
**Figure S14:** Funnel plot of the effect on the quantitative group.
**Figure S15:** Trim and fill plot for the effect on the quantitative group.

## Data Availability

Data sharing not applicable to this article as no datasets were generated or analysed during the current study.
