# Supplementary material for: HBV RNA Predicts the Risk of Off‐Treatment Relapse in Chronic Hepatitis B Patients With NAs Therapy: A Systematic Review and Meta‐Analysis
Source: J Viral Hepat. 2026 Mar 13;33(4):e70167. doi: 10.1111/jvh.70167 (PMC12988316; doi:10.1111/jvh.70167)
Supplement: Supplementary file 1 — Table S1: Search Strategy. S2 Table: Result of Newcastle‐Ottawa scale quality assessment. [file JVH-33-0-s001.docx]

S1 Table: Search Strategy

| Pubmed search strategy |  |
| --- | --- |
| 1 | Chronic Hepatitis B Virus Infection [Title/Abstract] |
| 2 | Chronic Hepatitis B [Title/Abstract] |
| 3 | Hepatitis B Virus Infection, Chronic [Title/Abstract] |
| 4 | Hepatitis B, Chronic [Title/Abstract] |
| 5 | hepatitis B virus pgRNA [Title/Abstract] |
| 6 | hepatitis B virus RNA [Title/Abstract] |
| 7 | HBV RNA [Title/Abstract] |
| 8 | HBV pgRNA [Title/Abstract] |
| 9 | hepatitis B virus pregenomic RNA [Title/Abstract] |
| 10 | HBV pregenomic RNA [Title/Abstract] |
| 11 | 1 or 2 or 3 or 4 |
| 12 | 5 or 6 or 7 or 8 or 9 or 10 |
| 13 | 11 and 12 |

S2 Table: Result of Newcastle-Ottawa scale quality assessment.

| Author Year | Total NOS score | Selection (4 scores) | | | | Comparability (2 scores) | Outcome (3 scores) | | |  |  |  |  |
| --- | --- | --- | --- | --- | --- | --- | --- | --- | --- | --- | --- | --- | --- |
|  |  | Representativeness of the exposed cohort (1 score) | Selection of the non-exposed cohort (1 score) | Ascertainment of exposure (1 score) | Outcomes were not present at study initiation (1 score) | Comparability of cohorts on the basis of ~~the~~ design or analysis | Assessment of outcome of follow-up (1 score) | Was follow-up long enough for outcome to occur (1 score) | Adequacy (1 score) |  |  |  |  |
| Apichat Kaewdech, 2020 [1] | 9 | 1 | 1 | 1 | 1 | 2 | 1 | 1 | 1 |  |  |  |  |
| Ivana Carey, 2020 [2] | | 8 | 1 | 1 | 1 | 0 | 2 | 1 | 1 | 1 |  |  |  |
| Rong Fan, Zhou B, 2020 [3] | 9 | 1 | 1 | 1 | 1 | 2 | 1 | 1 | 1 |  |  |  |  |
| Rong Fan, Peng J, 2020 [4] | 9 | 1 | 1 | 1 | 1 | 2 | 1 | 1 | 1 |  |  |  |  |
| Yayun Liu, 2020 [5] | 8 | 1 | 1 | 1 | 1 | 1 | 1 | 1 | 1 |  |  |  |  |
| Mireia García-López, 2021 [6] | 9 | 1 | 1 | 1 | 1 | 2 | 1 | 1 | 1 |  |  |  |  |
| Muye Xia, 2021 [7] | 9 | 1 | 1 | 1 | 1 | 2 | 1 | 1 | 1 |  |  |  |  |
| Wai-Kay Seto, 2021 [8] | | | 9 | 1 | 1 | 1 | 1 | 2 | 1 | 1 | 1 |  |  |
| Yandi Xie, 2021 [9] | 8 | 1 | 1 | 1 | 0 | 2 | 1 | 1 | 1 |  |  |  |  |
| Yue Liu, 2021 [10] | 8 | 1 | 1 | 1 | 0 | 2 | 1 | 1 | 1 |  |  |  |  |
| Apichat Kaewdech, 2022 [11] | 9 | 1 | 1 | 1 | 1 | 2 | 1 | 1 | 1 |  |  |  |  |
| Andreas Laras, 2022 [12] | 8 | 1 | 1 | 1 | 0 | 2 | 1 | 1 | 1 |  |  |  |  |
| Jingyu Chen 2022 [13] | 8 | 1 | 1 | 1 | 1 | 1 | 1 | 1 | 1 |  |  |  |  |
| Jinzhi He, 2022 [14] | | | | | 8 | 1 | 1 | 1 | 0 | 2 | 1 | 1 | 1 |
| Margarita Papatheodoridi, 2022 [15] | 8 | 1 | 1 | 1 | 0 | 2 | 1 | 1 | 1 |  |  |  |  |
| Alexander J. Thompson, 2023 [16] | | | | 8 | 1 | 1 | 1 | 0 | 2 | 1 | 1 | 1 |  |
| Florian van Bömmel 2024 [17] | 9 | 1 | 1 | 1 | 1 | 2 | 1 | 1 | 1 |  |  |  |  |
| Shuo Wu 2024 [18] | 8 | 1 | 1 | 1 | 1 | 1 | 1 | 1 | 1 |  |  |  |  |
| Simon J. Hume 2024 [19] | 8 | 1 | 1 | 1 | 1 | 1 | 1 | 1 | 1 |  |  |  |  |
| Norah A. Terrault 2024 [20] | 9 | 1 | 1 | 1 | 1 | 2 | 1 | 1 | 1 |  |  |  |  |
| Valerie Ohlendorf 2024 [21] | 7 | 1 | 1 | 1 | 0 | 2 | 1 | 0 | 1 |  |  |  |  |

NOS: Newcastle-Ottawa scale

Reference

1. Kaewdech, A., et al., Hepatitis B surface antigen, core-related antigen and HBV RNA: Predicting clinical relapse after NA therapy discontinuation. Liver Int 2020, 40 (12), 2961-2971.doi:10.1111/liv.14606

2. Carey, I., et al., Pregenomic HBV RNA and Hepatitis B Core-Related Antigen Predict Outcomes in Hepatitis B e Antigen-Negative Chronic Hepatitis B Patients Suppressed on Nucleos(T)ide Analogue Therapy. Hepatology 2020, 72 (1), 42-57.doi:10.1002/hep.31026

3. Fan, R., et al., Association Between Negative Results From Tests for HBV DNA and RNA and Durability of Response After Discontinuation of Nucles(t)ide Analogue Therapy. Clin Gastroenterol Hepatol 2020, 18 (3), 719-727.e7.doi:10.1016/j.cgh.2019.07.046

4. Fan, R., et al., Combining Hepatitis B Virus RNA and Hepatitis B Core-Related Antigen: Guidance for Safely Stopping Nucleos(t)ide Analogues in Hepatitis B e Antigen-Positive Patients With Chronic Hepatitis B. J Infect Dis 2020, 222 (4), 611-618.doi:10.1093/infdis/jiaa136

5. Liu, Y., et al., Serum HBV RNA Dynamic and Drug Withdrawal Predictor Value in Patients With Chronic HBV Infection on Long-term Nucleos(t)ide Analogue (NA) Therapy. J Clin Gastroenterol 2020, 54 (8), e73-e82.doi:10.1097/mcg.0000000000001376

6. García-López, M., et al., Viral and immune factors associated with successful treatment withdrawal in HBeAg-negative chronic hepatitis B patients. J Hepatol 2021, 74 (5), 1064-1074.doi:10.1016/j.jhep.2020.11.043

7. Xia, M., et al., Serum hepatitis B virus RNA level is associated with biochemical relapse in patients with chronic hepatitis B infection who discontinue nucleos(t)ide analogue treatment. Aliment Pharmacol Ther 2021, 54 (5), 709-714.doi:10.1111/apt.16538

8. Seto, W. K., et al., Role of serum HBV RNA and hepatitis B surface antigen levels in identifying Asian patients with chronic hepatitis B suitable for entecavir cessation. Gut 2021, 70 (4), 775-783.doi:10.1136/gutjnl-2020-321116

9. Xie, Y., et al., HBeAg-positive patients with HBsAg  < 100 IU/mL and negative HBV RNA have lower risk of virological relapse after nucleos(t)ide analogues cessation. J Gastroenterol 2021, 56 (9), 856-867.doi:10.1007/s00535-021-01812-0

10. Liu, Y., The research of predictive value of HBV RNA level on efficacy duration of NAs after discontinuation in CHB patients. 2021.doi:10.27749/d.cnki.gbzyx.2021.000106 (in Chinese)

11. Kaewdech, A., et al., Clinical Utility of SCALE-B to Predict Hepatitis B Virus Relapse, Hepatitis B Surface Antigen Loss After Antiviral Cessation in Asian Patients After 2-Year Follow-up. Front Med (Lausanne) 2022, 9, 859430.doi:10.3389/fmed.2022.859430

12. Laras, A., et al., Serum hepatitis B virus RNA detectability, composition and clinical significance in patients with ab initio hepatitis B e antigen negative chronic hepatitis B. Virol J 2022, 19 (1), 22.doi:10.1186/s12985-022-01749-7

13. CHEN Jing-yu , Q. W., HU Ming-le , LI Hua-dong , WANG Jing, Correlation between HBV pgRNA level and the risk of recurrence in patients with chronic hepatitis B after withdrawal of antiviral drug. Chinese Hepatology 2022, (27(04):413-417+425).doi:DOI:10.14000/j.cnki.issn.1008-1704.2022.04.004. (in Chinese)

14. He, J. Z., et al., [Clinical significance of hepatitis B virus pgRNA for deciding antiviral therapy discontinuation in patients with chronic hepatitis B]. Zhonghua Gan Zang Bing Za Zhi 2022, 30 (9), 970-975.doi:10.3760/cma.j.cn501113-20210305-00110

15. Papatheodoridi, M., et al., Significance of serum HBV RNA in non-cirrhotic HBeAg-negative chronic hepatitis B patients who discontinue effective antiviral therapy. J Viral Hepat 2022, 29 (11), 948-957.doi:10.1111/jvh.13729

16. Thompson, A. J., et al., Baseline serum HBV RNA is associated with the risk of hepatitis flare after stopping nucleoside analog therapy in HBeAg-negative participants. Hepatol Commun 2023, 7 (8).doi:10.1097/hc9.0000000000000188

17. van Bömmel, F., et al., Serum levels of hepatitis B core antibodies and hepatitis B core-related antigen at the time of nucleos(t)ide analog cessation and risk of severe flares in patients with chronic hepatitis B. Hepatol Commun 2025, 9 (3).doi:10.1097/hc9.0000000000000656

18. Wu, S., et al., Predictors of Nucleos(t)ide Analogues Discontinuation Relapse: Hepatitis B Virus RNA versus Hepatitis B Surface Antigen. J Coll Physicians Surg Pak 2024, 34 (5), 545-550.doi:10.29271/jcpsp.2024.05.545

19. Hume, S. J., et al., High end-of-treatment hepatitis B core-related antigen levels predict hepatitis flare after stopping nucleot(s)ide analogue therapy. Liver Int 2024, 44 (10), 2605-2614.doi:10.1111/liv.16029

20. Terrault, N. A., et al., Hepatitis B Virus RNA as a Biomarker for Safe Antiviral Discontinuation: A Prospective Study of Nucleos(t)ide Analogue Withdrawal. J Infect Dis 2025, 231 (5), 1290-1298.doi:10.1093/infdis/jiae541

21. Ohlendorf, V., et al., Limited Value of HBV-RNA for Relapse Prediction After Nucleos(t)ide Analogue Withdrawal in HBeAg-negative Hepatitis B Patients. J Viral Hepat 2025, 32 (4), e14026.doi:10.1111/jvh.14026
