# Supplementary material for: HBV RNA Predicts the Risk of Off‐Treatment Relapse in Chronic Hepatitis B Patients With NAs Therapy: A Systematic Review and Meta‐Analysis
Source: J Viral Hepat. 2026 Mar 13;33(4):e70167. doi: 10.1111/jvh.70167 (PMC12988316; doi:10.1111/jvh.70167)
Supplement: Supplementary file 2 — Figure S1: Subgroup analysis of the qualitative VR group: Depending on patients' different HBV HBeAg status. Figure S2: Subgroup analysis of the qualitative VR group: Depending on patients' different follow‐up duration. Figure S3: Subgroup analysis of the qualitative CR group: Depending on patients' different HBV HBeAg status. Figure S4: Subgroup analysis of the qualitative CR group: Depending on patients' different follow‐up duration. Figure S5: Subgroup analysis of the quantitative group: Depending on patients' different HBV HBeAg status. Figure S6: Subgroup analysis of the quantitative group: Depending on patients' different follow‐up duration. Figure S7: Sensitivity analysis of effect on the qualitative VR group‐ leave‐one‐out analysis. Figure S8: Sensitivity analysis of effect on the qualitative CR group‐ leave‐one‐out analysis. Figure S9: Sensitivity analysis of effect on the quantitative group‐leave‐one‐out analysis. Figure S10: Funnel plot of the effect on the qualitative VR group. Figure S11: Trim and fill plot for the effect on the qualitative VR group. Figure S12: Funnel plot of the effect on the qualitative CR group. Figure S13: Trim and fill plot for the effect on the qualitative CR group. Figure S14: Funnel plot of the effect on the quantitative group. Figure S15: Trim and fill plot for the effect on the quantitative group. [file JVH-33-0-s002.docx]

**Table of contents**

[Forest plot 1](#_Toc205564023)

[S1 Figure: Subgroup analysis of the qualitative VR group~~:~~ 2](#_Toc205564024)

[S2 Figure: Subgroup analysis of the qualitative VR group~~:~~ 3](#_Toc205564025)

[S3 Figure: Subgroup analysis of the qualitative CR group~~:~~ 4](#_Toc205564026)

[S4 Figure: Subgroup analysis of the qualitative CR group~~:~~ 5](#_Toc205564027)

[S5 Figure: Subgroup analysis of the quantitative group~~:~~ 6](#_Toc205564028)

[S6 Figure: Subgroup analysis of the quantitative group~~:~~ 6](#_Toc205564029)

[Sensitivity analyses 7](#_Toc205564030)

[Figure S7. Sensitivity analysis of effect on the qualitative VR group-leave-one-out analysis 7](#_Toc205564031)

[Figure S8. Sensitivity analysis of effect on the qualitative CR group- leave-one-out analysis 7](#_Toc205564032)

[Figure S9. Sensitivity analysis of effect on the quantitative group- leave-one-out analysis 8](#_Toc205564033)

[Funnel plots and “trim and fill” plots 8](#_Toc205564034)

[Figure S10. Funnel plot of the effect on the qualitative VR group 8](#_Toc205564035)

[Figure S11. Trim and fill plot for the effect on the qualitative VR group 9](#_Toc205564036)

[Figure S12. Funnel plot of the effect on the qualitative CR group 9](#_Toc205564037)

[Figure S13. Trim and fill plot for the effect on the qualitative CR group 10](#_Toc205564038)

[Figure S14. Funnel plot of the effect on the quantitative group 10](#_Toc205564039)

[Figure S15. Trim and fill plot for the effect on the quantitative group 11](#_Toc205564040)

# Forest plot

The squares demonstrate the weighted mean difference between intervention and the control groups. Different sizes of the squares illustrate the different weights of the studies’ sample sizes. The horizontal lines and parentheses demonstrate the 95% confidence interval.


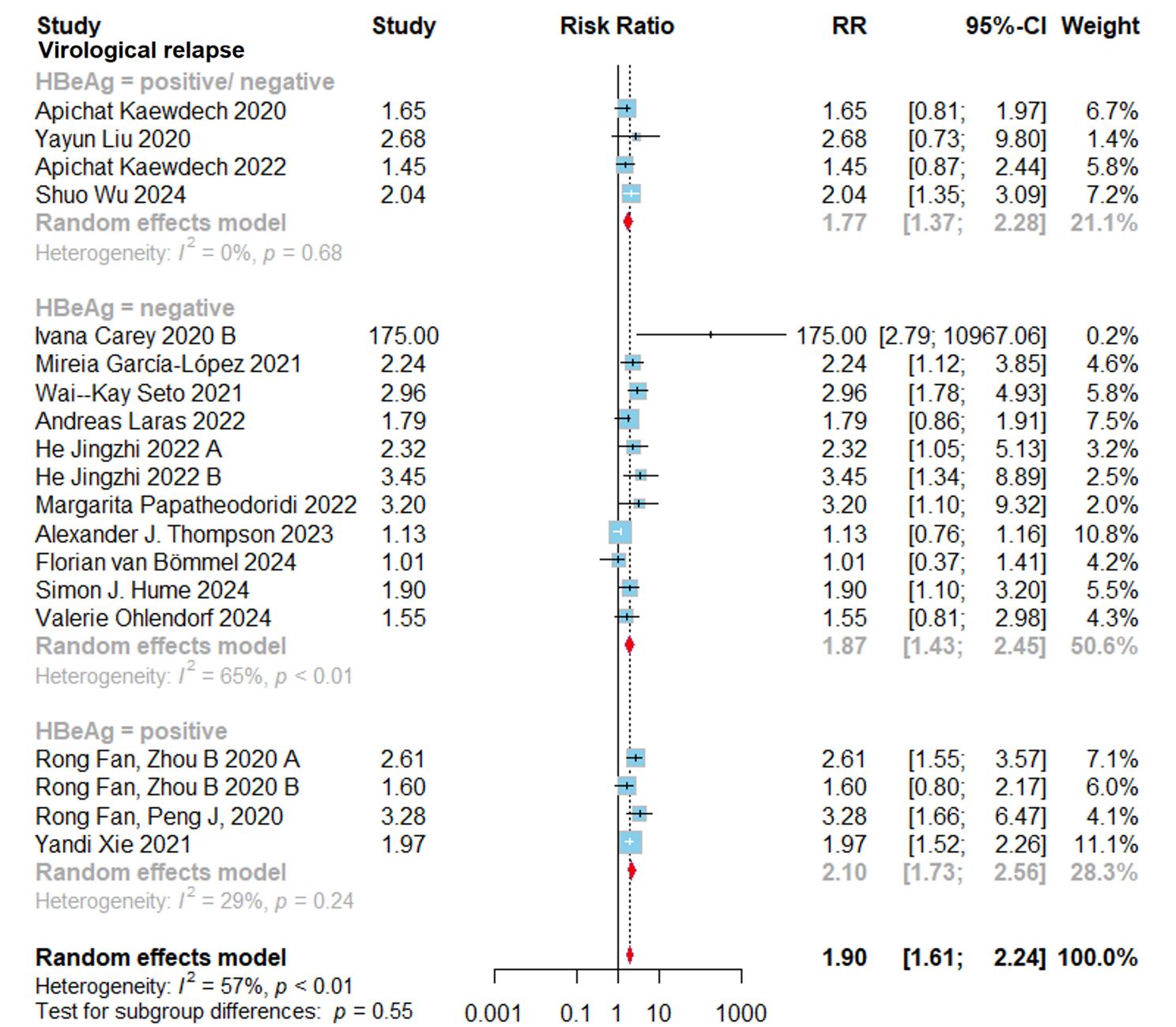


S1 Figure: Subgroup analysis of the qualitative VR group: **Depending on patients’ different HBV HBeAg status**

Based on 19 cohort studies. 95% CI is shown by horizontal lines and parentheses. CI: confidence interval; RR: risk ratio

**
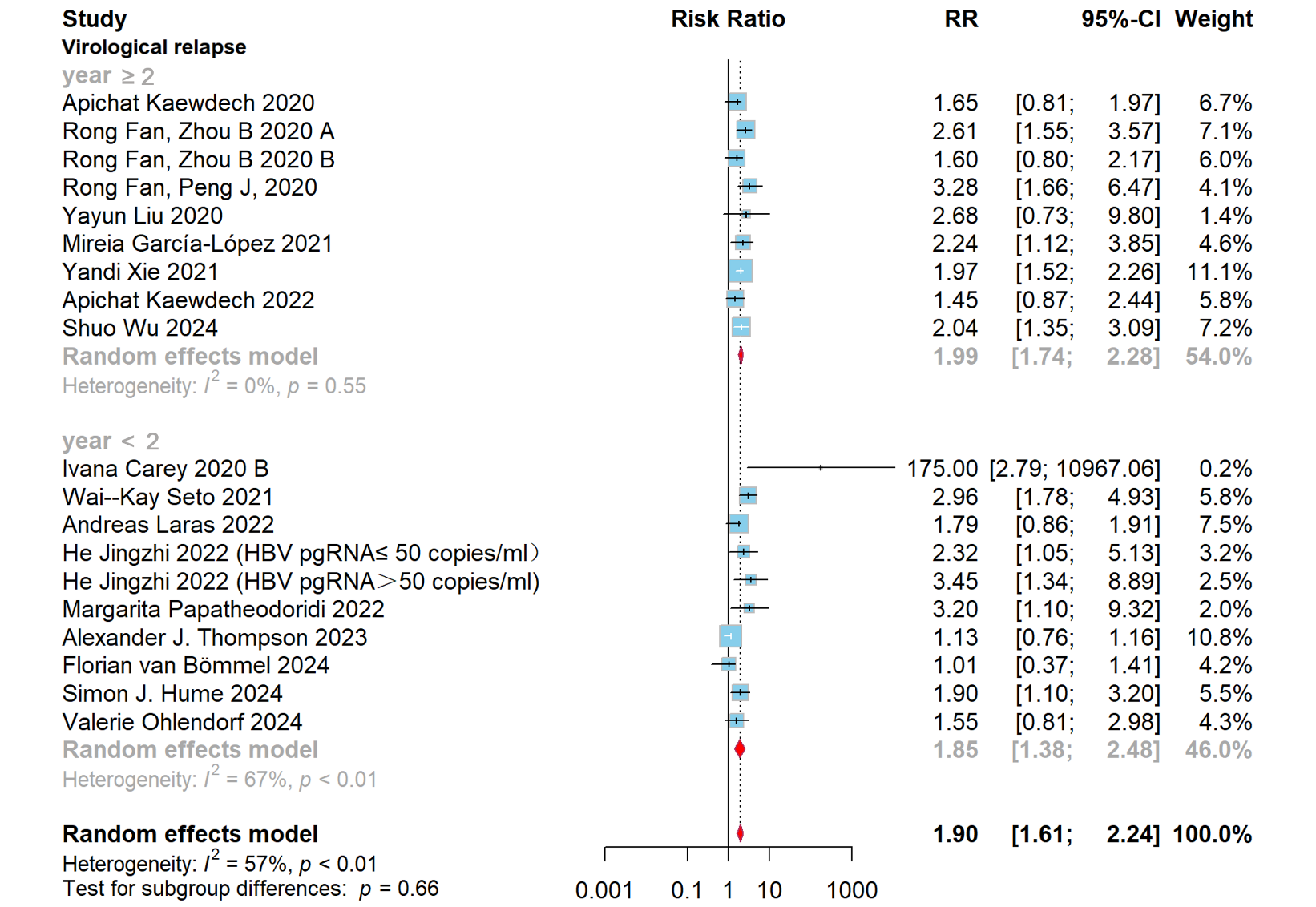
**S2 Figure: Subgroup analysis of the qualitative VR group: **Depending on patients’ different follow-up duration**

Based on 19 cohort studies. 95% CI is shown by horizontal lines and parentheses. CI: confidence interval; RR: risk ratio


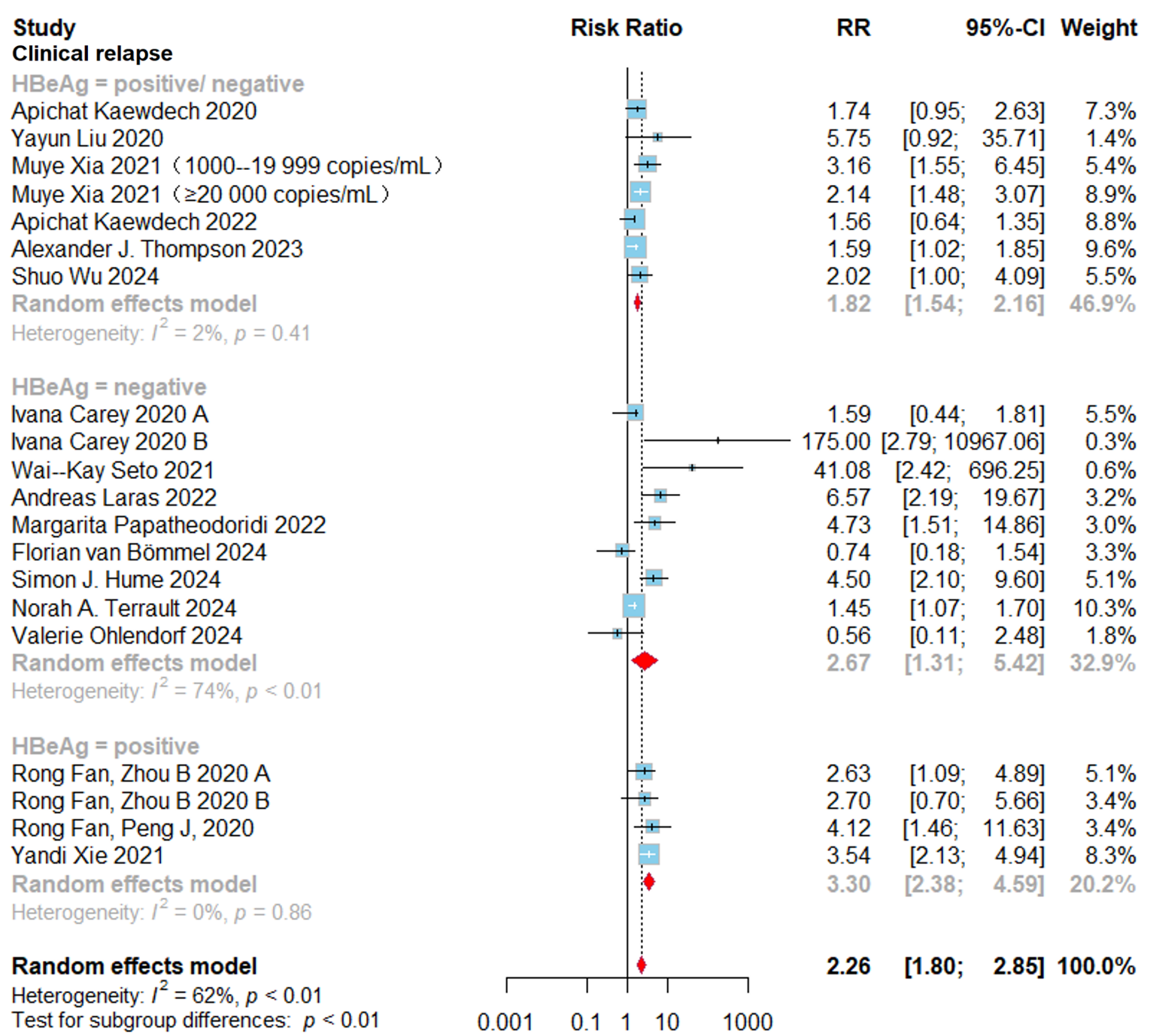
S3 Figure: Subgroup analysis of the qualitative CR group: **Depending on patients’ different HBV HBeAg status.**

Based on 20 cohort studies. 95% CI is shown by horizontal lines and parentheses. CI: confidence interval; RR: risk ratio.

**
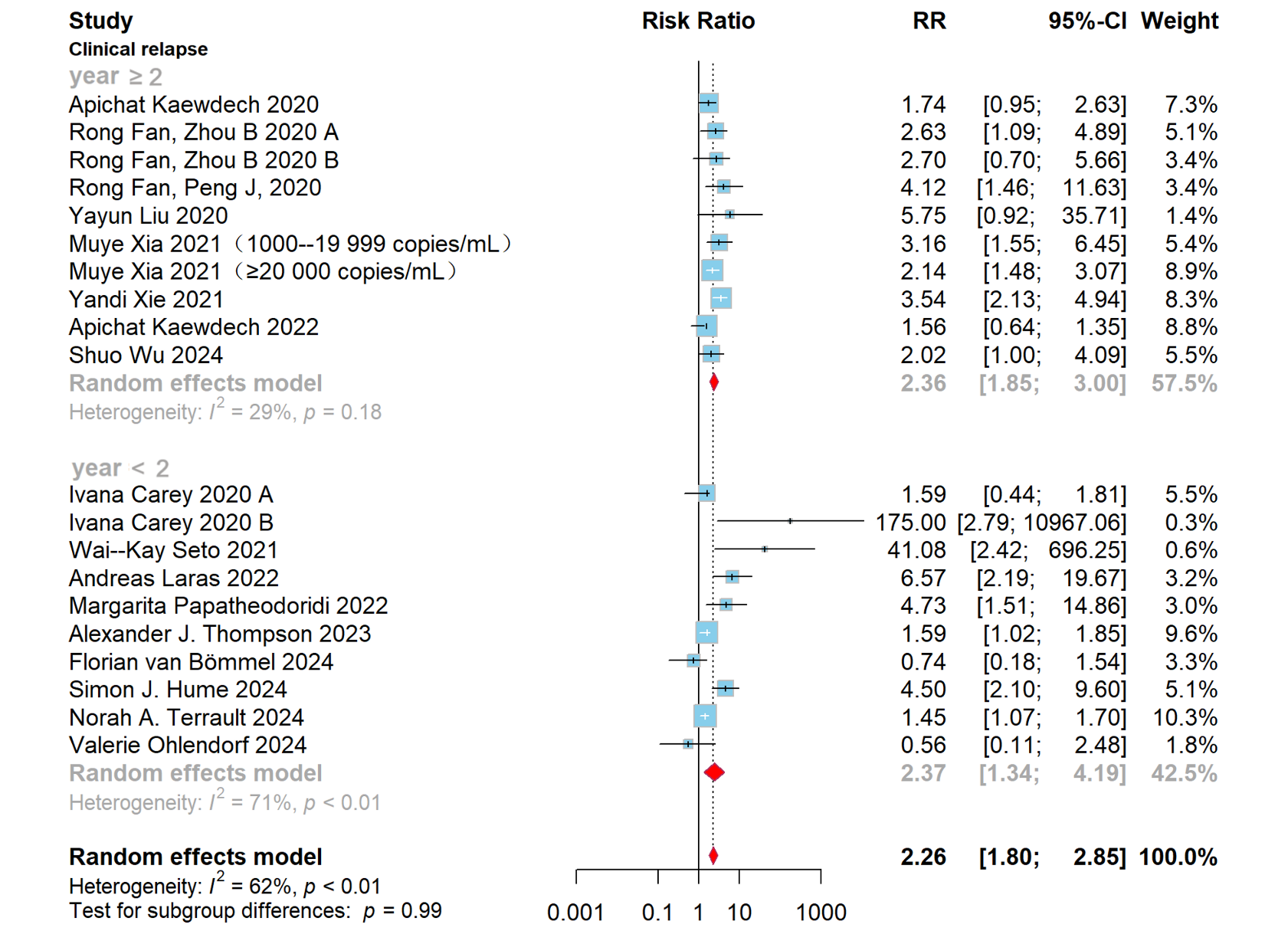
**S4 Figure: Subgroup analysis of the qualitative CR group: Depending on **patients’** different follow-up duration

Based on 20 cohort studies. 95% CI is shown by horizontal lines and parentheses. CI: confidence interval; RR: risk ratio.

**
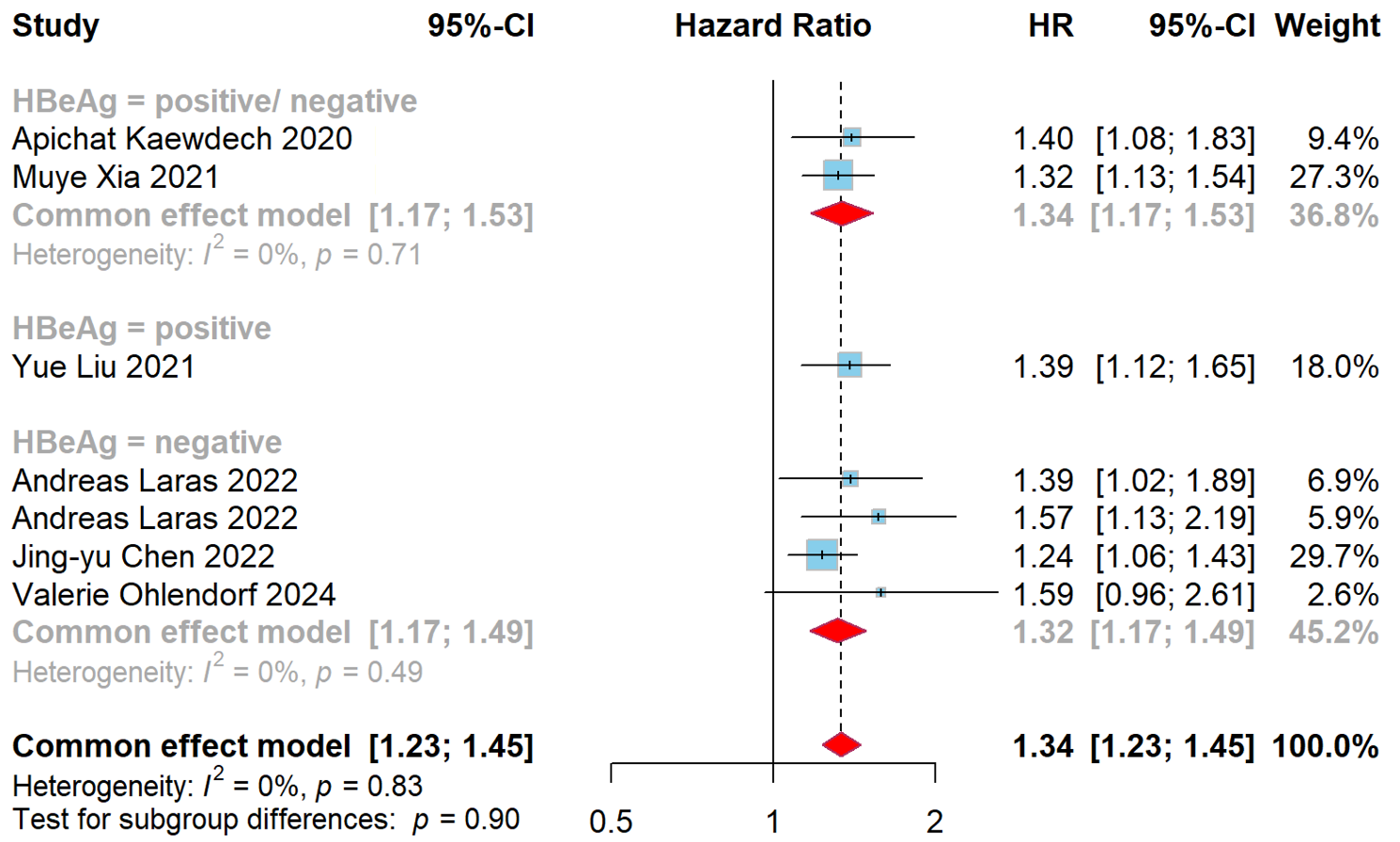
**S5 Figure: Subgroup analysis of the quantitative group: **Depending on patients’ different HBV HBeAg status.**

Based on 7 cohort studies. 95% CI is shown by horizontal lines and parentheses. CI: confidence interval; HR: Hazard Ratio.


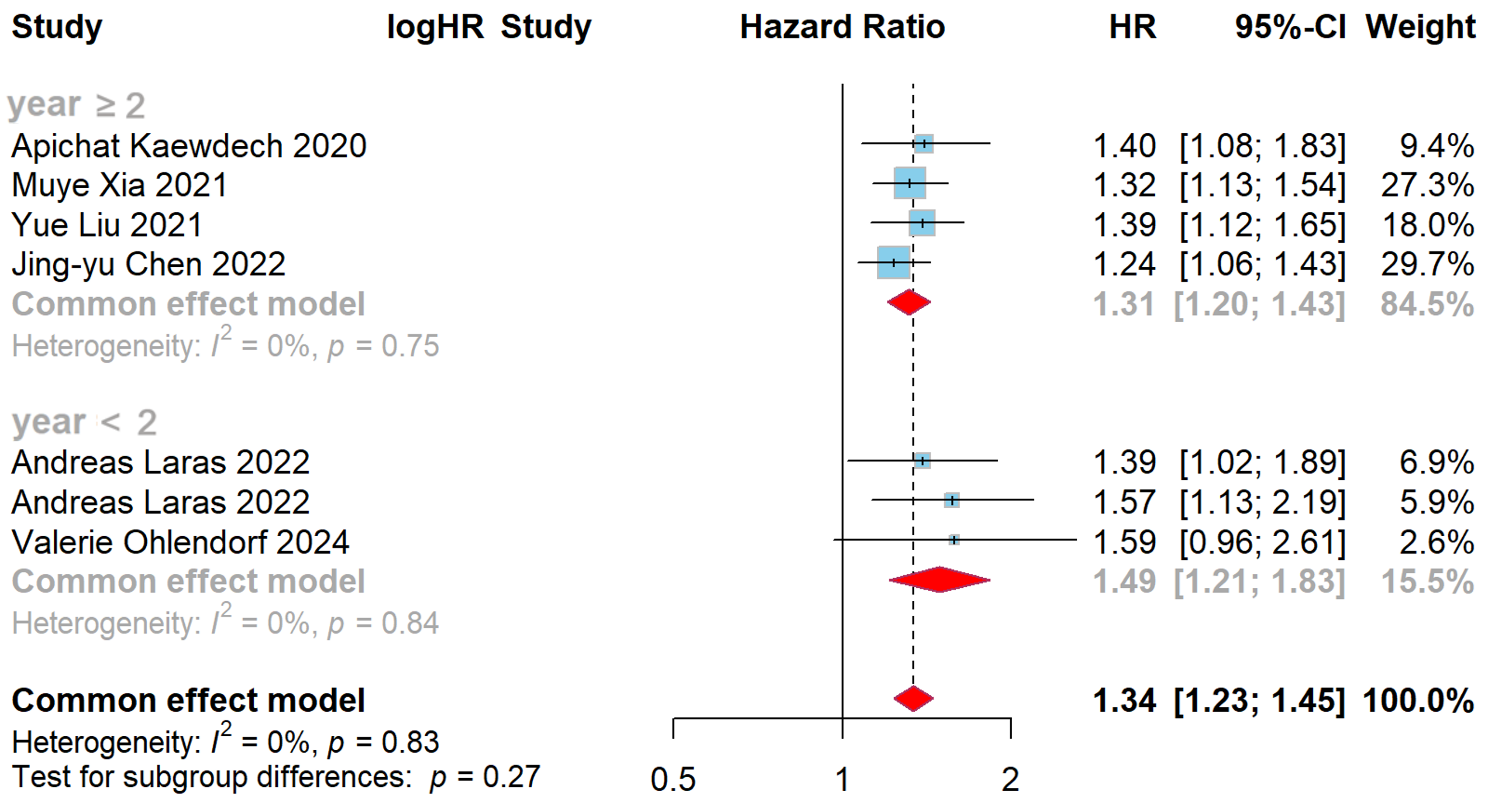


S6 Figure: Subgroup analysis of the quantitative group: **Depending on patients’** different follow-up duration

Based on 7 cohort studies. 95% CI is shown by horizontal lines and parentheses. CI: confidence interval; HR: Hazard Ratio.

# Sensitivity analyses


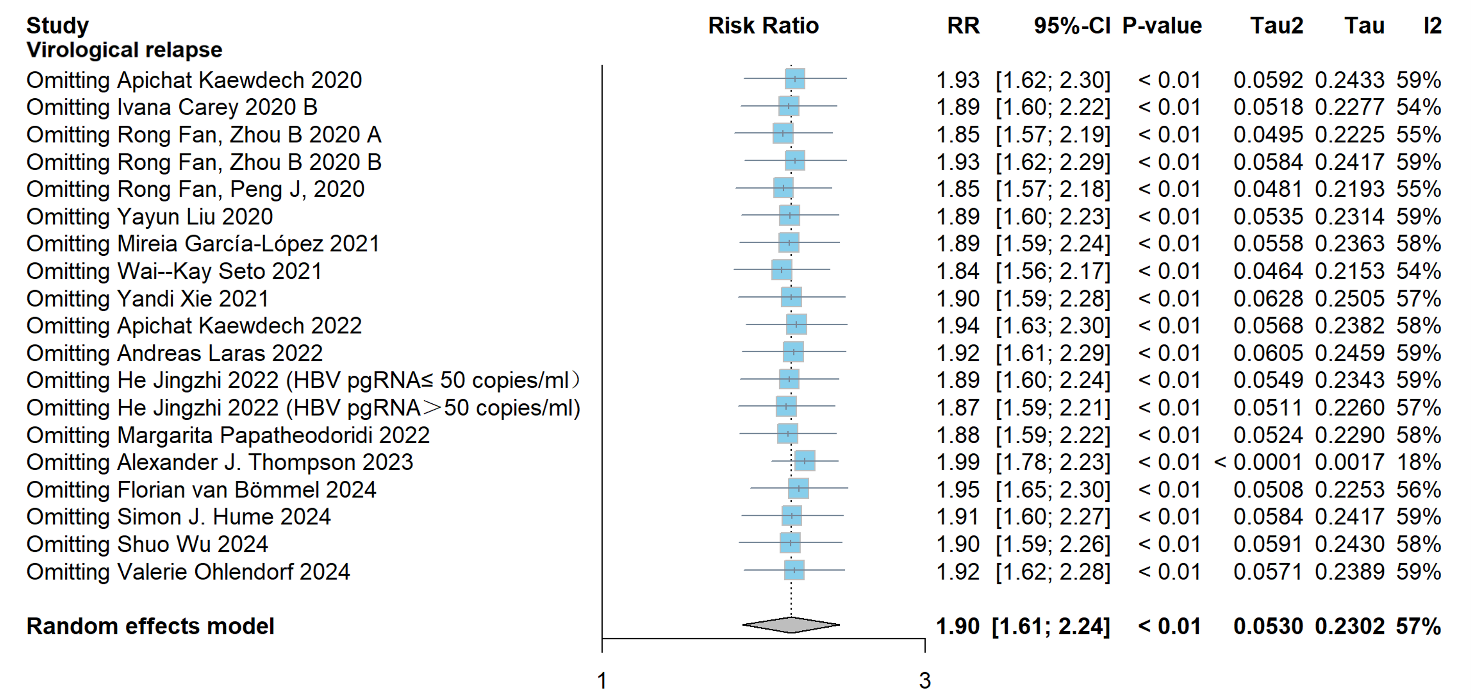
The blue boxes demonstrate the mean difference between intervention and the control groups. The horizontal lines and parentheses demonstrate the 95% confidence interval. The black dotted line demonstrates the overall mean effect size of the intervention. P-values indicate the significance of the effect estimate after removal of each study.

# Figure S7. Sensitivity analysis of effect on the qualitative VR group-leave-one-out analysis


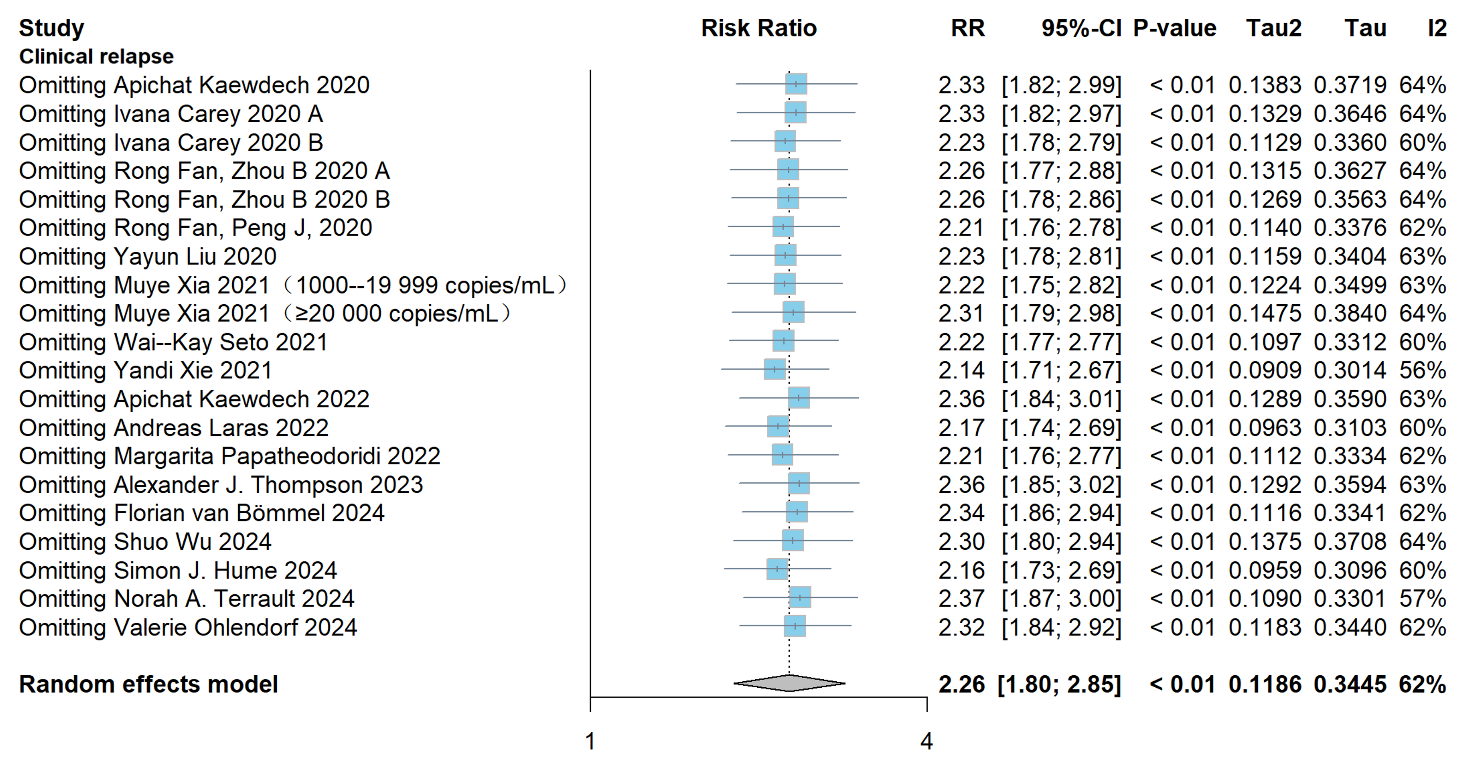
Based on 19 cohort studies. The studies indicated on the left are the studies excluded in the analysis. All *P* values =<0.001. CI: confidence interval.

## Figure S8. Sensitivity analysis of effect on the qualitative CR group- leave-one-out analysis

Based on 20 cohort studies. The studies indicated on the left are the studies excluded in the analysis. All *P* values =<0.001. CI: confidence interval.

##
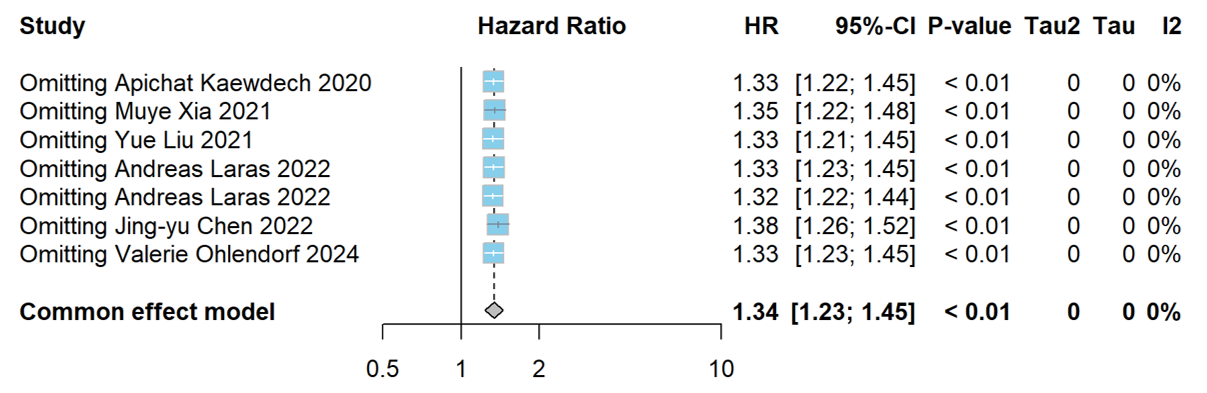
Figure S9. Sensitivity analysis of effect on the quantitative group-leave-one-out analysis

Based on 7 cohort studies. The studies indicated on the left are the studies excluded in the analysis. All *P* values =<0.001. CI: confidence interval.

# Funnel plots and “trim and fill” plots

The y-axis is the standard error of the effect. The x-axis is the mean difference between intervention and the control group. Each dot demonstrates the mean effect estimate of one study. Studies with larger sample sizes are on the top, while studies with smaller sample sizes are in the lower region of the plot. The black dotted line represents the 95% confidence interval, while the black dotted, vertical line illustrates the estimated overall effect of the intervention. The empty circle dots in the trim and fill plots are imputed studies to achieve plot symmetry.


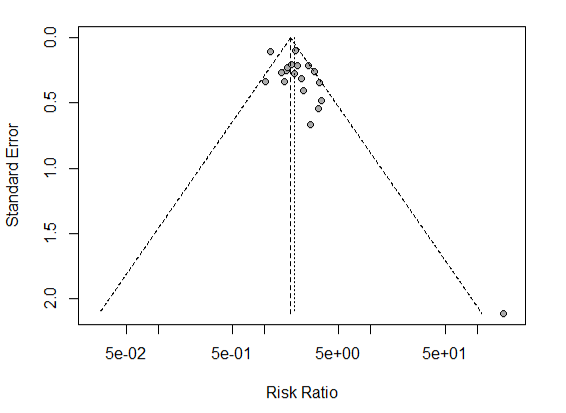


Based on 19 cohort studies.

## Figure S10. Funnel plot of the effect on the qualitative VR group

##
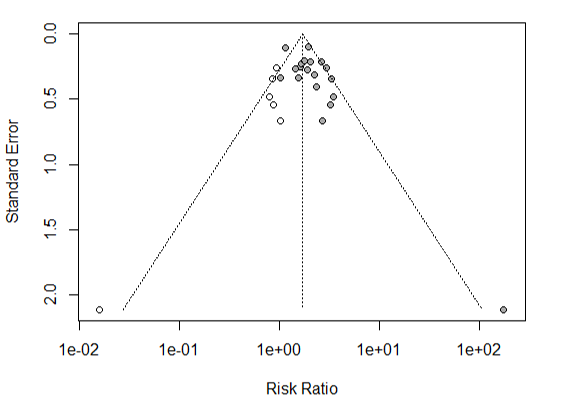
Figure S11. Trim and fill plot for the effect on the qualitative VR group

Based on 25 studies: 19 cohort studies

and 6 imputed trials.


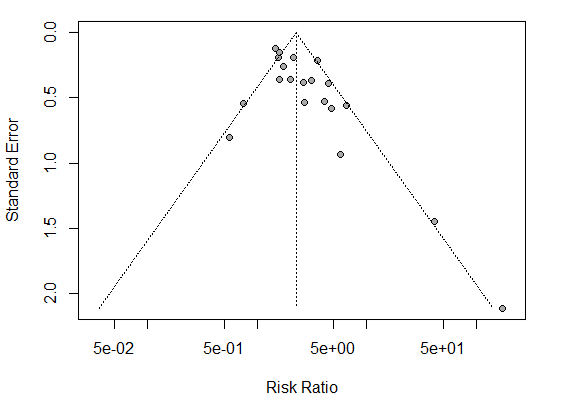


## Figure S12. Funnel plot of the effect on the qualitative CR group

Based on 20 studies.

##
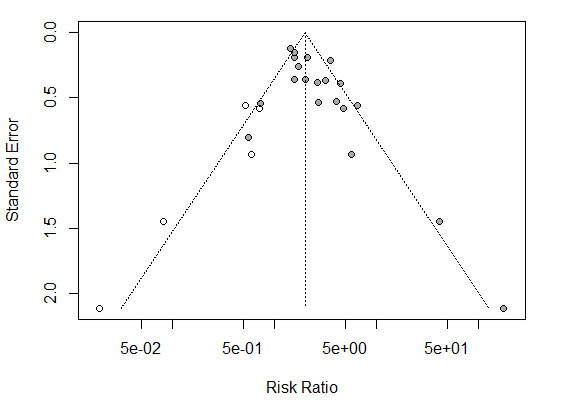
Figure S13. Trim and fill plot for the effect on the qualitative CR group

Based on 25 studies: 20 cohort studies

and 5 imputed trials.

**
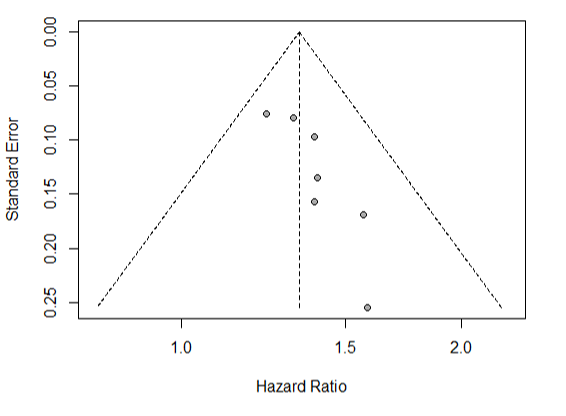
**

## Figure S14. Funnel plot of the effect on the quantitative group

Based on 7 studies.

Based on 7 cohort studies.

##
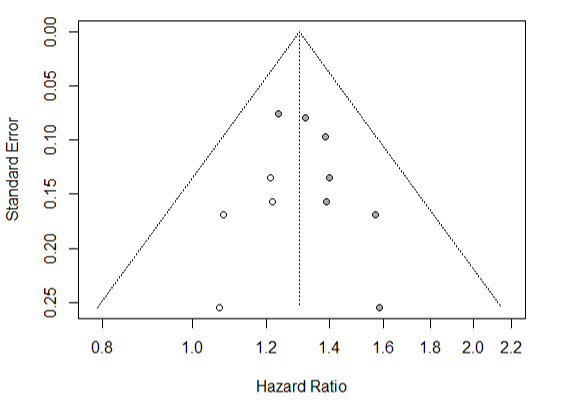


## Figure S15. Trim and fill plot for the effect on the quantitative group

Based on 11 studies: 7 cohort studies

and 4 imputed trials.
